# Supplementary material for: Cardiovascular risk factors and outcomes in COVID-19: A hospital-based study in India
Source: PLOS Glob Public Health. 2022 Apr 1;2(4):e0000234. doi: 10.1371/journal.pgph.0000234 (PMC10021757; doi:10.1371/journal.pgph.0000234)
Supplement: Attachment — Submitted filename: PLOSGH_RTR_Covid_CVRisk.docx [file pgph.0000234.s002.docx]

**Ref: PGPH-D-21-01016**

Original article entitled: Cardiovascular risk factors and outcomes in COVID-19: a hospital-based study in India.

**Response to Reviewers**

**Reviewer #1:**

This is a large single-centre study exploring predictors COVID-19 outcomes in hospitalised patients. Whilst many such studies have already been published, this is the largest such study from India and likely one of the largest from a LMIC.

**A: Thanks for the encouragement.**

The specific aim was to explore the association of cardiovascular risk factors with COVID-19 outcomes. The rationale for restricting consideration to cardiovascular risk factors only is unclear.

**A: Previous studies show that cardiovascular risk factors are an important determinant of out comes in Covid-19. This has been made more clear in Introduction section, para 2.**

Why not consider all the variables that the authors collected data for - for example they have data on pulmonary disease?

**A: The number of patients with established cardiovascular disease and pulmonary disease were low (Figure 1).**

Also, how was the cut-off for age selected? A binary cut-off of 50 years greatly limits the information we can take from the analysis. Age could either be used as a continuous variable, or could be categorised in to multiple levels (e.g. <40, 49-59, 60-69, 70+).

**A: Thanks for the comment. We have now divided age groups into three, <40, 40-59 and >60. Results show an escalating trend in cardiovascular risk factors, deaths and other outcomes with age. The Abstract, Methods, Results and Conclusion sectiona have been revised accordingly. Table 1 has also been revised. Age is used as a continuous variable for multivariate adjustments.**

For Table 6 it would be better to present the 'full' adjusted models as separate tables - i.e. one table per model showing all the factors adjusted for - this would make it much easier to interpret.

**A: Thanks. We have now been added to Methods section that deaths is the primary outcome and other outcomes are secondary. Accordingly Results section has been modified. Tables 6 and 7 separately present primary and secondary outcomes.**

There is some lack of clarity around whether the authors were aiming to describe outcomes in these groups (i.e. based on unadjusted analysis only) or to determine whether these are independent risk factors (i.e. based on adjusted analysis). A factor may have a significant association with an outcome on an unadjusted analysis, but after adjusting for possible confounders there is no longer a significant association (i.e. patients with this factor have higher risk of the outcome, but it isn't actually because of this factor but due to some other confounder). This is largely what has happened in this study. Whilst all the CV variables are associated with mortality, in table 6 only age is an independent predictor of mortality. Similarly, only hypertension is an independent predictor of invasive ventilation and only age is a independent predictor of non-invasive ventilation. Seeing the full models would help with interpretation of this. So, although the authors state that "this study shows that older patients, males, and those with hypertension, diabetes and any tobacco use have greater risk of deaths and adverse outcomes from COVID-19" it is not true that all these factors are independently associated with those outcomes.

**A: We agree that age is the most important risk factor. However, it is well known that all the cardiovascular risk factors increase with age and there is significant interaction among them. We believe that the focus should be on cardiovascular risk factors (important on univariate analyses) with age associated associations showing attenuation of risk (as seen on age and sex adjusted analyses). We believe that this should be the primary results. Total attenuation of risk with multivariate analyses show that there is a significant interaction of age and risk factors with clinical presentation and outcomes. These observations have been added to discussion section.**

Other comments

1. Please make use of a relevant reporting guideline and complete the appropriate checklist, e.g. STROBE

**A: Added.**

2. Please define a single primary outcome and secondary outcomes

**A: Added to Abstract, Methods and Results**

3. The authors describe this as a prospective study. Does this mean that patients were prospectively identified and outcomes collected at the point of hospital discharge?

**A: Yes. However, we have simplified the sub-title to: Hospital Based Study in India.**

4. Were children included - this is not mentioned

**A: Thanks for the comment. Now added to Abstract, Methods and Results sections.**

**Reviewer #2:**

The study methodology is not quite clear although the author indicated a prospective study in the title; it was not appreciated in the methodology. For a prospective study, features such as duration of the study and time of outcomes should be specified which was not done, author only stated the period for enrolment. If indeed a prospective design was employed, a parameter of interest to the author such as BMI could have been measured. I think the study used an existing database of patients containing registry of case files and medical history hence reporting on suspected Covid-19 cases was not necessary since the study sought to look for outcomes in Covid-19 and not outcomes in suspected Covid-19.

**A: Thanks. Yes, it is essentially a registry-based cross-sectional study and all consecutive patients of Covid-19 have been included.**

The author should give information on Covid-19 (confirmed by RT-PCR) since it was readily available in the hospital registry.

**A: Added to the Methods section. All the patients included were RT-PCR test positive Covid-19.**

Finally, no sample size was calculated for the study so I assumed every unit of the population was captured.

**A: Yes, it is essentially a registry-based study design and all consecutive patients have been enrolled.**

The author should be more modest about presentation of methodology, apart from these observations the study is good.

**A: Thanks.**
